# Growth condition dependency is the major cause of non-responsiveness upon genetic perturbation

**DOI:** 10.1371/journal.pone.0173432

**Published:** 2017-03-03

**Authors:** Saman Amini, Frank C. P. Holstege, Patrick Kemmeren

**Affiliations:** 1 Princess Máxima Center for Pediatric Oncology, Utrecht, The Netherlands; 2 Center for Molecular Medicine, University Medical Centre Utrecht, Utrecht, The Netherlands; Texas A&M University College Station, UNITED STATES

## Abstract

Investigating the role and interplay between individual proteins in biological processes is often performed by assessing the functional consequences of gene inactivation or removal. Depending on the sensitivity of the assay used for determining phenotype, between 66% (growth) and 53% (gene expression) of *Saccharomyces cerevisiae* gene deletion strains show no defect when analyzed under a single condition. Although it is well known that this non-responsive behavior is caused by different types of redundancy mechanisms or by growth condition/cell type dependency, it is not known what the relative contribution of these different causes is. Understanding the underlying causes of and their relative contribution to non-responsive behavior upon genetic perturbation is extremely important for designing efficient strategies aimed at elucidating gene function and unraveling complex cellular systems. Here, we provide a systematic classification of the underlying causes of and their relative contribution to non-responsive behavior upon gene deletion. The overall contribution of redundancy to non-responsive behavior is estimated at 29%, of which approximately 17% is due to homology-based redundancy and 12% is due to pathway-based redundancy. The major determinant of non-responsiveness is condition dependency (71%). For approximately 14% of protein complexes, just-in-time assembly can be put forward as a potential mechanistic explanation for how proteins can be regulated in a condition dependent manner. Taken together, the results underscore the large contribution of growth condition requirement to non-responsive behavior, which needs to be taken into account for strategies aimed at determining gene function. The classification provided here, can also be further harnessed in systematic analyses of complex cellular systems.

## Introduction

Understanding the interplay between individual proteins and their role in various biological processes is critical for understanding cellular systems as well as understanding genotype to phenotype relationships. The role of individual proteins is often investigated by assessing the functional consequences of inactivation, often through genetic perturbation. With the advent of efficient gene editing techniques such as CRISPR-Cas9, such reverse genetics approaches are now starting to be even more expansively applied [1]. Pioneering work by the yeast gene deletion consortium using homologous recombination has in the past yielded an exhaustive collection of gene deletion mutants for *Saccharomyces cerevisiae* [2]. Subsequent high-throughput studies have enabled systematic investigation of the functional consequences of deleting individual genes and have been conducted to investigate cellular systems in many different ways [3–6]. However, a large number of deletion strains do not show detectable phenotypic effects and the scale and cause of this non-responsive phenomenon has been the subject of considerable interest [7–14]. In *S*. *cerevisiae* over 66% of deletion mutants have little or no detectable effect on growth in a single rich medium [2,15]. Similar observations have been made for other organisms [16,17]. Growth is not necessarily the most sensitive assay and as an alternative, genome-wide gene expression can be used, also as a much more detailed phenotypic readout. A recent study investigating the consequences of almost 1,500 gene deletions revealed that 53% of these mutants have a genome-wide expression profile that is essentially the same as wildtype (WT) [3]. This confirms that a large proportion of gene deletions have no phenotype when assayed under a single condition. Lack of a phenotype is due to either redundancy mechanisms or due to growth condition dependency of the deleted gene. An important question addressed insufficiently so far, is the degree to which these different mechanisms contribute to non-responsiveness. Understanding the underlying causes and their relative contribution is important for understanding complex cellular systems and for improving reverse genetic strategies aimed at determining gene function.

There are at least two kinds of redundancy mechanisms that contribute to non-responsive behavior upon genetic perturbation. One factor is the presence of closely related paralogs (homology-based redundancy) [7,10,11,14]. Redundancy can also be achieved through the presence of alternative pathways that can facilitate the same biological process (pathway-based redundancy) [11,18]. Homology-based redundancy is attributed to gene duplicates or proteins with similar functional domains. Even though most close paralog pairs have diverged during evolution [19], there are still pairs that have retained the capability to compensate for each other’s loss [10,11,14,20]. Previous estimates indicate that as much as 23% to 29% of non-responsiveness can be ascribed to duplicated genes [7,10]. These estimates are however based on growth-rate as the phenotypic read-out and involved analysis of only a single growth condition. It therefore remains to be seen whether these estimates are similar when applying a more sensitive phenotypic read-out and taking into account multiple growth conditions. To what degree pathway-based redundancy contributes to non-responsiveness is still unclear. This is mainly due to the fact that the degree to which negative genetic interactions reflect buffering between two genes in parallel pathways has remained unsolved. In addition to negative genetic interactions between parallel pathways, many negative genetic interactions have been detected between seemingly unrelated pathways [21–23]. It has also been suggested that most negative genetic interaction scores are not due to simple redundancy between pathways, but rather reflect network topology [24,25]. Providing clear estimates of the contribution of pathway-based redundancy to non-responsiveness solely based on negative genetic interactions is therefore unsatisfactory. Additional sources of information likely need to be included in order to provide reliable estimates of pathway-based redundancy.

Besides various redundancy mechanisms, condition dependency is another factor that contributes to non-responsiveness upon genetic perturbation. Some genes are almost certainly only required under specific growth conditions. It has been suggested that a large number of seemingly non-responsive mutants have important fitness roles under specific environmental conditions [8,18,26]. These estimates vary between 37% and 97%, using either focused subsets of genes, different assays or a combination of heterozygous and homozygous deletion strains. The degree to which these different approaches affect condition dependency estimates is unclear. A uniform analysis, using a single resource of non-responsive deletion strains is therefore required for reliably estimating the contribution of condition dependency to non-responsiveness, also in combination with redundancy estimates.

In spite of much effort, the causes and mechanisms of non-responsiveness remains elusive [7,11,18,26]. Most studies have either focused on a single explanation or have insufficiently investigated the underlying mechanisms and their relative contributions. Here, using a sensitive phenotypic readout (gene expression) as a starting point, we systematically investigate the underlying causes of and their relative contribution to non-responsiveness upon gene deletion. This reveals that condition dependency is the major determinant of non-responsiveness, explaining approximately 71%. Homology-based and pathway-based redundancy contributes 17% and 12% respectively. For approximately 14% of protein complexes, just-in-time assembly provides a potential mechanistic explanation for how protein complexes can be regulated in a condition dependent manner. The classification of the relative contribution of underlying causes provided here, can be harnessed for other systematic analyses of cellular systems.

## Results

### A collection of non-responsive mutants

To systematically investigate the underlying causes of and their relative contribution to non-responsiveness observed upon gene deletion, we exploited the availability of a collection of 1,484 deletion mutants expression profiles in the yeast *Saccharomyces cerevisiae* [3]. To obtain the list of responsive and non-responsive mutants, the same definitions were applied as in the original study [3]. In short, a mutant is considered non-responsive (NR) if three or less transcripts are changing significantly (FC > 1.7 & *p*-value < 0.05) as a consequence of deleting a particular gene. A mutant is classified as responsive (R) if four or more transcripts are changing significantly upon gene deletion. Applying these thresholds, 784 mutants are classified as NR (53%) and 700 mutants are classified as R (47%) as indicated before [3].

**Fig 1:**
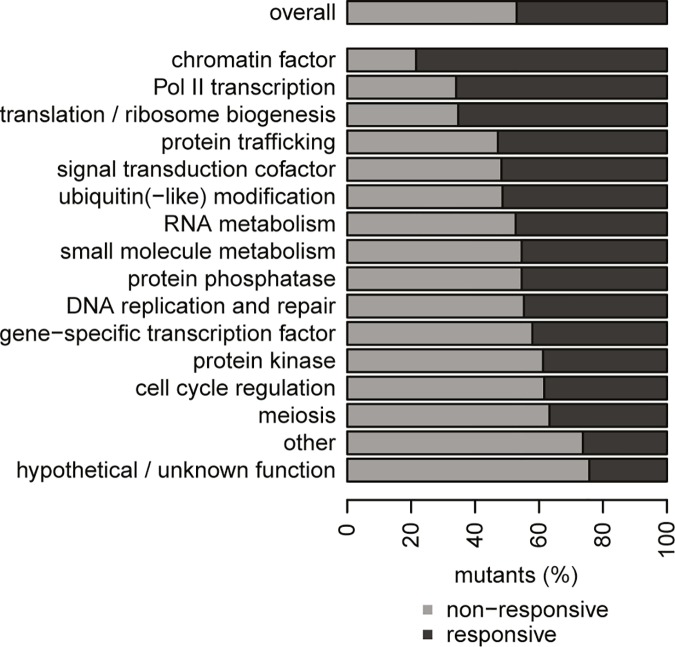
Distribution of non-responsive behavior. Percentage of non-responsive (NR; light grey) and responsive (R; dark grey) mutants within different functional categories, as well as the overall percentage. A mutant is classified as NR if three or less transcripts are changing compared to WT. A mutant is classified as R if four or more transcripts are changing. Functional categories as defined in the original study [3].

To ensure that non-responsiveness is not restricted to a particular class of proteins, the relative contribution of non-responsiveness to different functional classes was investigated [3]. As expected, non-responsiveness is not limited to a specific functional class, but common to a wide range of functional classes (Fig 1). There are differences however in the relative contribution of non-responsiveness to various functional classes. Classes that have a central role in cellular systems such as chromatin factors, Pol II transcription and translation / ribosome biogenesis have a relatively low number of NR mutants. Other functional classes such as cell cycle regulation and meiosis have a high number of NR mutants. This indicates that there is a relationship between the relative importance of different functional classes and degree of non-responsiveness, but also suggests that different causes such as condition dependency (meiosis) and robustness (cell cycle regulation) may contribute to non-responsiveness.

### Both redundancy and condition dependency contribute to non-responsiveness

**Fig 2:**
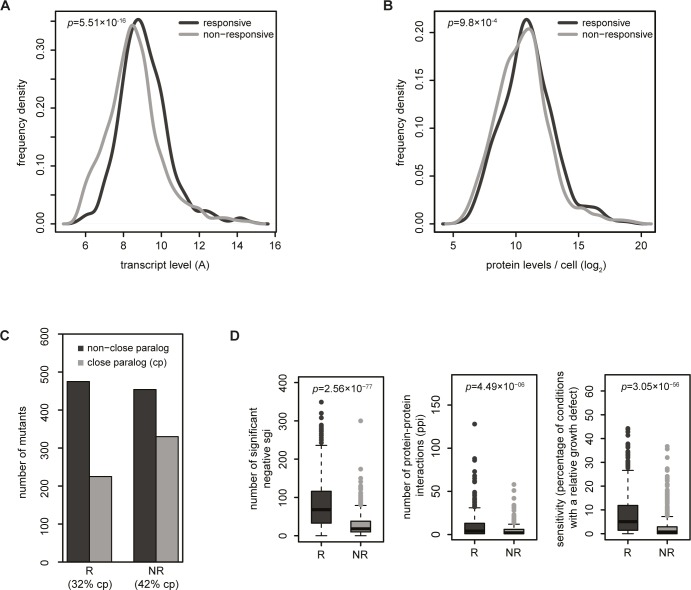
General characteristics of non-responsive and responsive mutants. **(A)** Frequency density distribution of wildtype (WT) mRNA transcript levels (A) obtained from the average of 200 WT strains, adapted from Kemmeren et al. [3]. *P*-value indicates the difference between transcript levels in the WT strains between NR and R mutants based on a two-sided Mann-Whitney test. **(B)** Frequency density distribution of the number of proteins per WT cell [27] for NR and R mutants, adapted from Kemmeren et al. [3]. Only detectable proteins are depicted. *P*-value indicates the difference between protein levels in NR and R mutants based on a two-sided Mann-Whitney test. **(C)** Number of NR and R mutants with a close paralog (CP) that arose from whole-genome duplication (WGD) [31] or small scale duplications (SSD) [20]. **(D)** Box plots showing the number of significant negative synthetic genetic interaction (SGI) scores [22](ε ≤ -0.08, *p* ≤ 0.05; left panel), number of protein-protein interactions [30](PPI, middle panel) and sensitivity to different conditions [8] (percentage of conditions that deletion mutants show a relative growth defect; growth defect > 0, *p* ≤ 0.05; right panel) for NR and R mutants. *P*-values are based on a two-sided Mann-Whitney test.

Exclusive requirement of a gene under a specific growth condition is expected to contribute to the number of non-responsive mutations. As a starting point, differences in mRNA transcript and protein levels between responsive and non-responsive mutants were investigated. As also observed before [3], genes within the group of NR mutants have a slightly larger fraction of genes with low transcript [3] and protein [27] levels (Fig 2A and 2B). This also reveals that almost all genes within the group of NR mutants generally exhibit normal expression levels in wildtype (WT), both for transcript as well as protein levels. Their removal however, has little effect on gene expression and raises the question why so many genes are expressed while not being required under the condition investigated.

Another likely cause of non-responsiveness is redundancy. This is confirmed by a strong enrichment of close paralogs in NR mutants (Fig 2C). Interestingly, NR mutants exhibit markedly less negative genetic interactions [22] (Fig 2D, left panel). Negative genetic interactions do not necessarily reflect redundancy [10,11,24,28,29]. It is also suggested that redundant gene pairs have few negative genetic interactions as they are only expected to show a defect when both genes are absent [10], just as indicated here. NR mutants also have fewer physical interactions [30] and show less growth defects when assayed across many different conditions [8] (Fig 2D, middle and right panel). This is all confirmatory of more specialized, less centrally required roles in cellular biology and that both redundancy and condition dependency are contributing causes of non-responsiveness.

### Close paralogs as a proxy for homology-based redundancy

**Fig 3:**
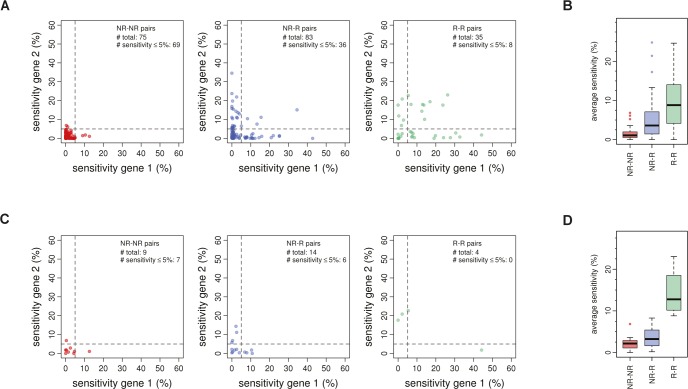
Homolog-based redundancy estimates. **(A)** Scatter plots depicting the sensitivity of gene 1 and gene 2 for close paralog pairs between non-responsive (NR-NR, left panel), between non-responsive and responsive (NR-R, middle panel) or between responsive (R-R, right panel) mutants. **(B)** Boxplots of the average sensitivity of the same groups as in A. The difference between all three groups is statistically significant (*p*-value _(NR-NR vs. NR-R)_ = 3.14×10^−9^; *p*-value _(NR-NR vs. R-R)_ = 1.02×10^−8^; *p*-value _(NR-R vs. R-R)_ = 1.66x10^-3^) based on a one-sided Mann-Whitney test. **(C)** Same as in A, except that only close paralog pairs with a significant negative SGI score are shown. **(D)** Boxplots of the average sensitivity of the same groups as in C (*p*-value _(NR-NR vs. NR-R)_ = 0.128, *p*-value _(NR-NR vs. R-R)_ = 1.4x10^-3^, *p*-value _(NR-R vs. R-R)_ = 3.27x10^-3^), statistical test as in B.

To provide a reliable estimate of the relative contribution of complete redundancy to non-responsiveness, close paralog pairs within the non-responsive mutants are used here. Close paralog pairs are among the most recent evolutionary duplications and probably still have a similar function [32,33]. Close paralog pairs that show non-responsive behavior are therefore most likely the best starting point for estimating the relative contribution of redundancy to non-responsiveness. If two genes form a complete redundancy pair, it is also expected that removing either one of these genes does not result in any measurable defect, regardless of the condition investigated. The dataset from Hillenmeyer et al. [8] measures the relative growth defect of individual deletions in many different conditions. Pairs with a complete redundancy relationship are expected to have low sensitivity to different environmental conditions as measured in this dataset (Materials and Methods). Indeed, close paralog pairs between non-responsive mutants exhibit low sensitivity to different environmental conditions (Fig 3A, left panel). On the other hand, close paralog pairs between non-responsive and responsive mutants or between responsive mutants show a significant increase in sensitivity to different conditions (Fig 3A, middle & right panel; Fig 3B). This effect is stronger when only considering negative genetic interactions within the pairs depicted in Fig 3A (Fig 3C and 3D), confirming that negative genetic interactions provide useful additional information when investigating redundancy.

Interestingly, a large difference in sensitivity between the various close paralog groups is observed (Fig 3A and 3B), suggesting that there might be a disparity in sequence divergence among the close paralog pairs. The number of non-synonymous substitutions per non-synonymous site (Ka) is often considered an indicator of sequence divergence [14]. Although there seems to be a slight difference in the Ka values for close paralog pairs between non-responsive (NR-NR), between non-responsive and responsive (NR-R) or between responsive mutants (R-R), this is not significant (S1A Fig). When considering percentage sequence identity, a significant difference is only observed when using the domains shared between close paralog pairs (Materials and Methods; S1B and S1C Fig). This effect is lost when using all pairs but close paralog pairs (S1D Fig), suggesting that compensation capability is conserved between pairs with the same ancestral gene.

To ensure that homology-based redundancy estimates are reliable, several sensitivity cutoffs and their corresponding false-discovery rate (FDR) were investigated (Materials and Methods). Selecting close paralog pairs between non-responsive mutants with 5% or less sensitivity leads to the lowest FDR (≈ 10%; S2 Fig). Based on this, the contribution of homology-based redundancy to non-responsiveness is estimated at approximately 17% (Fig 3A, left panel; S1 Table). This encompasses 133 unique genes out of 784 NR mutants that are likely non-responsive due to a complete redundancy relationship with another gene.

### Negative genetic interactions indicate pathway-based redundancy

**Fig 4:**
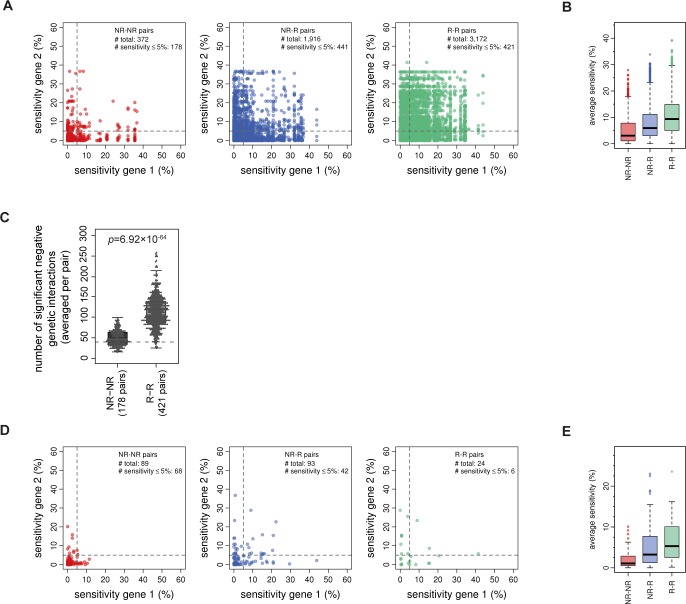
Pathway-based redundancy estimates. **(A)** Scatter plots depicting the sensitivity of gene 1 and gene 2 for pairs with a significant negative genetic interaction score between non-responsive (NR-NR), between non-responsive and responsive (NR-R) or between responsive (R-R) mutants. Close paralog pairs are excluded. **(B)** Boxplots of the average sensitivity of the same groups as in B, all three are significantly different (*p*-value < 2.2x10^-16^). *P*-values are based on a one-sided Mann-Whitney test. **(C)** Boxplot showing the number of significant negative genetic interactions for NR-NR and R-R pairs with 5% or less sensitivity. Close paralog pairs are excluded. The dashed line indicates the threshold (40) taken to obtain an FDR of 8%. **(D)** Scatter plots shown for the same pairs in A that in addition have, on average, less than 40 negative genetic interactions per pair. **(E)** Boxplots of the average sensitivity of the same groups as in D (*p*-value _(NR-NR vs. NR-R)_ = 1.31×10^−7^, *p*-value _(NR-NR vs. R-R)_ = 3.64×10^−6^, *p*-value _(NR-R vs. R-R)_ = 0.064), statistical test as in B.

In addition to homology-based redundancy (Fig 3A and 3B), where two genes can directly compensate for each other’s loss, two genes can also operate in two redundant pathways. Negative genetic interactions have in the past been associated with redundant pathways [34]. Pairs of non-responsive mutants with negative genetic interactions show less sensitivity to different conditions compared to pairs between non-responsive and responsive or between responsive mutants (Fig 4A and 4B), as also observed before for close paralog pairs (Fig 3). However, taking into account the 5% sensitivity cutoff for estimating the contribution of pathway-based redundancy, the FDR rate is much higher (approximately 70%) compared to homology-based redundancy estimates. This clearly indicates that only using negative genetic interactions as a proxy for pathway redundancy is insufficient for estimating the contribution of pathway-based redundancy to non-responsiveness.

If two genes operate in a completely redundant manner, they should only exhibit a negative genetic interaction score with each other and not with any other gene [10]. Since the removal of a single gene can be completely compensated by the other gene, there should be no measurable phenotypic effect, except when both genes are deleted. The number of negative genetic interactions is indeed significantly lower for pairs between non-responsive mutants compared to responsive mutants (Fig 4C). Using a cutoff on the number of negative genetic interactions (40), a similar degree of reliability (FDR ≤ 10%) as obtained for homology-based redundancy estimates can be obtained (Fig 4D and 4E; Materials and Methods). Pathway-based redundancy estimates were therefore based on pairs between non-responsive mutants that fulfilled four criteria. First, pairs are not close paralogs of each other. Second, pairs have a significant negative genetic interaction. Third, pairs have 40 or less negative genetic interactions with other genes. And last, pairs show, on average, 5% or less sensitivity in other conditions. In total, 68 pairs, 97 unique genes, can be associated with pathway-based redundancy (Fig 4D, left panel; S2 Table), indicating that approximately 12% (FDR ≈ 8%) of non-responsiveness can be attributed to pathway-based redundancy.

### Relative contribution of redundancy and condition dependency to non-responsiveness

**Fig 5:**
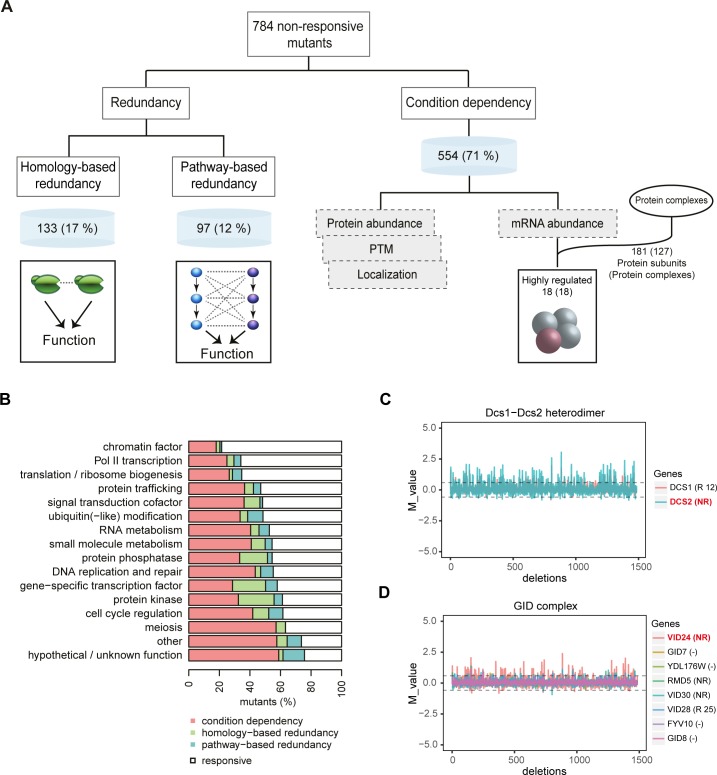
Relative contribution of redundancy and condition dependency and potential mechanisms. **(A)** Flowchart showing the relative contribution of redundancy and condition dependency to non-responsiveness. Potential mechanisms are indicated for homology-based redundancy, pathway-based redundancy and “just-in-time assembly”. **(B)** Relative contribution of redundancy and condition dependency to non-responsiveness within each functional category. **(C)** Line plot showing the mRNA expression changes across 1,484 deletion mutants [3] for the Dcs1-Dcs2 heterodimer. Individual lines indicate the Dcs1 and Dcs2 subunit. The highly regulated subunit is indicated in red. **(D)** Line plot as in C for the GID complex.

By combining the estimates of homology-based redundancy (133 genes, 17%) and pathway-based redundancy (97 genes, 12%), the overall contribution of redundancy to non-responsiveness is approximately 29% (Fig 5A). This number is a rough estimate and the exact percentage also depends on the cutoffs used. Here, stringent cutoffs are used to avoid a high FDR, but therefore potentially interesting pairs might be missed that do not fulfill the stringent criteria. Nevertheless, the analysis indicates that the majority of non-responsiveness, as much as 71%, is attributable to condition dependency (554 genes; Fig 5A; S3 Table). Interestingly, different functional classes do show diverse behavior in the relative contribution of condition dependency and redundancy (Fig 5B). Non-responsiveness for classes such as meiosis, DNA replication and repair have a relatively high contribution of condition dependency. A few classes such as gene-specific transcription factors, protein kinases and protein phosphatases however, show a much higher contribution of homology-based redundancy, indicating that the degree of redundancy differs between different functional classes (Fig 5B).

Mechanistic explanations for homology-based as well as pathway-based redundancy have been proposed before [28,29]. For homology-based redundancy, this is based on two proteins that can completely replace each other’s function (Fig 5A). For instance, the redundant kinases Ark1 and Prk1 that both phosphorylate the same consensus target amino acid sequence and are involved in regulating endocytosis and actin skeleton [24,35]. Another example includes Upc2 and Ecm22, two redundant gene-specific transcription factors that bind to the same set of target genes and regulate ergosterol biosynthesis [36,37]. A final example involves Nhp6a and Nhp6b, two highly homologous HMGB proteins involved in modulation of chromatin structure [38]. These examples are all indicative of more direct relationships for homology-based redundancy pairs with proteins having the ability of taking over the exact same function.

For pathway-based redundancy, the concept is that two pathways or proteins can compensate each other’s function in a more indirect fashion (Fig 5A). Although the exact relationship between many of these pairs is still unclear, they are often found to be involved in the same process and loss of both leads to significant unexpected defects. One example is the relationship between Ecm1 and Pom34. Ecm1 is a pre-ribosomal factor involved in pre-60S particle export and Pom34 is a subunit of the nuclear pore complex (NPC). In the absence of Pom34, depletion of Ecm1 leads to defects in pre-60S particle export [39]. Another example includes Kar5 and Prm3, which are both involved in nuclear membrane fusion during karyogamy and loss of both genes leads to nuclear fusion defects [40]. Note that, as highlighted with these cases, the underlying mechanism for pairs associated with pathway-based redundancy is often unclear.

### Potential mechanisms for condition dependent genes

The mechanistic explanations provided for redundancy are useful in understanding the interplay between different proteins and how this relates to cellular processes. For genes/proteins required only under certain conditions, regulation can occur at many different levels, such as regulation of mRNA and protein levels, post-translational modifications (PTMs) that change aspects of protein-protein interactions and cellular localization. Many genes that are non-responsive still have relatively high mRNA and protein expression levels (Fig 2A and 2B), suggesting that these are constitutively expressed despite not being required under the given condition. For cell-cycle related protein complexes, De Lichtenberg et al. [41] have proposed a mechanism termed “just-in-time assembly” to indicate that most members of these protein complexes are constitutively expressed and only a few key subunits need to be regulated in order to control the activity of the entire protein complex. Many proteins are expressed under the condition investigated here, but do not result in a detectable phenotypical defect when removed. This therefore raises the question of whether “just-in-time assembly” may also apply to condition dependency.

To investigate this, all proteins that are part of a protein complex were obtained using a predefined definition of protein complexes [42]. Out of 554 genes attributed to condition dependency 181 are part of 127 unique protein complexes (Fig 5A). To determine the degree of differential regulation within each protein complex, highly regulated subunits were detected based on the degree of variation in expression levels across different deletion mutants (Material and Methods). Out of the 127 protein complexes, 18 complexes contain a highly regulated subunit (≈14%; Fig 5A; S3 Fig). To ensure that these results are not limited to the subset of proteins analyzed here, the same analysis was also performed using all protein coding genes in yeast. This revealed that 69 protein complexes out of 501 protein complexes [42] contain a highly regulated subunit (≈14%), confirming the results obtained when using the non-responsive mutants attributed to condition dependency.

One example of a protein complex with a highly regulated subunit includes the Dcs1-Dcs2 heterodimer that removes capped mRNA fragments left over from mRNA decay [43]. Previous reports indicated that Dcs2 is most sensitive to transcriptional induction and suppresses Dcs1 activity in response to nutrient stress [44,45]. Here, Dcs2 is identified as a highly regulated subunit (Fig 5C), confirming its role as a modulator of the activity of the Dcs1-Dcs2 heterodimer. Another example includes the GID complex, consisting of seven subunits [46–48]. The GID complex targets the key enzyme fructose-1,6-biphosphatase (FBPase) for proteosomal degradation during the switch from gluconeogenesis to glycolysis [48]. Vid24, the regulatory subunit of the GID complex, initiates FBPase polyubiquitination and subsequent degradation [48]. Vid24 protein levels are undetectable under gluconeogenic conditions, but rapidly accumulate when glucose is added and decrease together with FBPase [48]. Our analysis also reveals Vid24 as a highly regulated subunit (Fig 5D), consistent with previous reports about the role of Vid24 in regulating the activity of the GID complex [46,48]. Besides these known examples, there are also putative protein complexes that have a highly regulated subunit (S3 Fig) that can be used as a starting point for further unraveling their function and potential regulatory mechanism. Taken together, approximately 14% of protein complexes are regulated similar to the “just-in-time assembly” model, but in a condition-dependent manner.

## Discussion

### Redundancy estimates

The high proportion of gene deletions that fail to exhibit a detectable defect under a specific condition has muddled efforts aimed at systematic phenotyping. Elucidating gene function requires the ability to elicit a detectable phenotypical readout, such as a growth defect or gene expression changes. Over 66% of deletion mutants in *Saccharomyces cerevisiae* have no detectable growth phenotype [15] and over 53% of non-essential gene deletions hardly show any gene expression changes [3]. Understanding the underlying causes of this non-responsive behavior will aid in elucidating gene function and understanding the complexity of cellular systems. Here, we estimate that approximately 29% of non-responsiveness can be attributed to either homology-based (17%) or pathway-based (12%) redundancy. Previous estimates have indicated that between 23% and 29% of negative genetic interactions can be ascribed to duplicated genes [7,10]. Our estimate of 17% is most likely lower because we require low sensitivity under multiple conditions (Fig 3A) whereas previous estimates are based on growth under a single condition. For complete redundancy pairs, deletion of a single gene should exhibit no detectable phenotype under any given condition. It is therefore expected that the redundancy pairs that we miss, but are included in previous estimates will fail to exhibit a redundancy relationship when investigated under different conditions.

### Homology-based redundancy versus pathway-based redundancy

Estimates for the contribution of redundancy to non-responsiveness include both homology-based as well as pathway-based redundancy. The known examples found for pathway-based redundancy indicate a higher degree of uncertainty as they are found to be involved in the same process and loss of both leads to significant defects. The exact mechanism however, is often unknown. Since there is no direct sequence-based evidence for a redundancy relationship between these pairs, it remains to be seen to what degree pathway-based redundancy relationships can fully compensate the loss of one gene, or whether this is only partial. One can also question whether these should be considered as redundancy relationships, or whether these most likely reflect pathway connectivity or combinatorial effects as already suggested before [24,28]. The most likely candidate pairs providing redundancy are therefore those based on homology (estimated at 17%), whereas most other negative genetic interactions reflect pathway connectivity.

### Partial redundancy

Partial redundancy is also expected to contribute to non-responsive behavior. Simplistically, two different types can be differentiated. For the first type, only one of the two genes involved elicits a response under the condition investigated. The other type consists of a pair whereby deletion of either gene does not result in a detectable phenotype under the condition investigated. However, at least one of the genes would elicit a response when removed under a different (environmental) condition. We deliberately did not include these pairs within our redundancy estimates, as the purpose here is to investigate to what degree complete redundancy contributes to non-responsive behavior. Pairs that can fully compensate for each other’s loss are expected to do so given any circumstance. It is therefore much more likely that partial redundancy is either an intrinsic property of pathway connectivity, just as for many pathway-based redundancy pairs, or is a different way to achieve condition dependency. The relationship might then indicate a manner to regulate condition dependency or ensure that genes with a similar function, but required under different environmental conditions can behave in a switch-like fashion so that the most efficient protein is available under the right circumstances.

### Condition dependency and just-in-time assembly

While condition dependency can be regulated in many different ways, we focused here on a mechanism shown previously for cell cycle related protein complexes [41]. The just-in-time assembly mechanism may provide a mechanistic explanation how protein complexes can be regulated in a condition-dependent manner. Regulation of approximately 14% of all protein complexes can be explained in such a way. This still leaves many protein complexes unexplained and indicates that the just-in-time assembly is not such a widespread mechanism. Interestingly, many of the protein complexes with a highly regulated subunit are involved in metabolism. Almost 50% of these protein complexes are associated with metabolism and this percentage is even higher for heterodimers. Metabolic flux analyses have shown that the activity of metabolic pathways can be regulated in different ways and through several regulatory factors [49]. Although not necessarily the most predominant mode of action, transcriptional regulation of key regulatory factors has been shown as one way to achieve condition- and tissue-specific control of metabolic pathway activity [50]. The enrichment of heterodimers also fits the idea of enzymatic protein complexes consisting of a regulatory and catalytic subunit. The mechanism found here, might thus be more specific for metabolic pathways and protein complexes involved in metabolism.

The term just-in-time assembly proposed by De Lichtenberg et al. [41] to describe the dynamic formation of protein complexes during the cell cycle might not always be appropriate for the highly regulated subunits found here. The term implies that the entire complex is assembled just before it is needed. This doesn’t necessarily need to be the case. For instance, most of the protein complex could already be pre-assembled and only the regulatory subunit joins the partly pre-assembled protein complex when needed. Since it is impossible to assess the exact mechanism by which a protein complex is activated using only gene expression data, we propose to use “just-in-time activation” as a more general term. This also allows other types of just-in-time activation to be included, such as protein phosphorylation or other post-translational modifications. Additional data exploring condition dependent protein expression or post-translational modifications are needed to gain more detailed mechanistic insights.

## Materials and methods

### Gene expression data and functional categories

The list of deletion mutants and corresponding gene expression profiles were obtained from Kemmeren et al [3]. Each gene expression profile consists of *p*-values and average mRNA transcript changes (M values; log_2_(fold-change)) relative to wildtype (WT). Genes are considered significantly changed when fold-change (FC) > 1.7 and *p*-value < 0.05. Genes that were frequently changing regardless of the deletion mutant (WT variable genes) were excluded as described in the original study [3]. The number of differentially expressed transcripts was used to classify mutants either as responsive (R) or non-responsive (NR) as done previously [3]. Mutants with less than 4 transcript changes relative to WT are considered NR and mutants with 4 or more changes are considered R. Mutants were grouped in different functional categories as before [3]. Both the overall ratio of NR and R mutants as well as for each individual functional category (Fig 1) was calculated.

### mRNA transcript levels in WT

A wild-type pool consisting of 200 WTs [3] was used to obtain average mRNA transcript levels (A values; log_2_(R*G)) for all genes corresponding to the different deletion mutants. mRNA transcript levels in WT were compared between NR and R mutants. *P*-values are calculated using a Mann-Whitney two-sided test to evaluate differences in mRNA transcript levels between NR and R mutants (Fig 2A).

### Protein expression levels, protein-protein interactions and protein complexes

The number of protein molecules per WT cell was obtained from Ghaemmaghami et al. [27]. Proteins with undetectable signals, low signals or experimental problems were excluded. Protein expression levels were compared between NR and R mutants. *P*-values are calculated using a Mann-Whitney two-sided test to evaluate differences in protein levels between NR and R mutants (Fig 2B). Protein-protein interactions were obtained from Collins et al. [30]. The number of protein-protein interactions between NR and R mutants was compared. *P*-values are calculated using a Mann-Whitney two-sided test (Fig 2D, middle panel). Protein complexes (501) were obtained from the curated ‘‘consensus + GO” set from Benschop et al. [42].

### Close paralog genes

For close paralog genes, both small scale duplications (SSD) as well as whole-genome duplications (WGD) are used. A list of SSD pairs was obtained from Guan et al. [20]. WGD (547 pairs) were obtained from Byrne and Wolf [31]. Global protein sequence alignment for each close paralog pair was performed using the Needleman-Wunsch algorithm [51] as implemented in Needle-EMBOSS version 6.6 [52] with default options. Only SSD pairs with 20% or higher identity were kept (751 pairs). Combining both WGD and SSD resulted in a total of 1,298 close paralog pairs. Close paralog pairs for which both genes were profiled [3] were used for further downstream analyses (205 pairs).

### Genetic interaction data

Synthetic genetic interaction (SGI) scores were obtained from Costanzo et al. [22]. SGI scores were acquired for all pairwise combinations of NR and R mutants for which a score was calculated (Figs 3 and 4). For determining significant negative genetic interactions, the same cutoff was applied as used originally [22] (ε ≤ -0.08, *p* ≤ 0.05).

### Condition sensitivity

Growth rate of yeast homozygous gene deletions compared against WT under multiple conditions (418 experiments) was obtained from Hillenmeyer et al. [8]. A relative growth rate above zero is an indication of a growth defect of a deletion mutant relative to WT in a particular condition. A two-sided t-test was applied to assign *p*-values. Growth rates from duplicate conditions were averaged, resulting in 278 unique conditions. Only significant growth defects were selected for downstream analysis (*p* ≤ 0.05). The sensitivity of each gene is expressed as the percentage of significant relative growth defects within the 278 unique conditions.

### Sequence divergence

The number of non-synonymous substitutions per non-synonymous site (Ka) was calculated based on a method introduced by Yang and Nielsen [53]. This method is implemented in the GenomeHistory 2.0 tool, which was used to calculate Ka values [54]. Non-default parameters chosen include: minimum translated ORF: 100; minimum number of aligned residues to accept pair: 100; accepting all BLAST hits with e < = 1e-08 and minimum percentage identity for analysis: 40% (S1A Fig). Protein sequence identity for close paralog pairs was calculated either across the whole protein sequence or only for protein domains using the Needleman-Wunsch algorithm [51] as implemented in Needle-EMBOSS version 6.6 [52] with default options (S1B Fig). InterProscan version 5.10–50 [55] was used to obtain a list of shared domains between genes. Only protein domains identified by Pfam [56] were included in downstream analyses. Sequence identity was calculated between two shared Pfam domains using the same tool as used for complete protein sequences. Gene pairs with at least one shared Pfam domain were included in the downstream analysis. Sequence identity for Pfam domains with the same identifier were averaged before averaging all distinct shared domains across two proteins.

Averages of sequence identity between shared Pfam domains for gene pairs were compared either for close paralog or non-paralog pairs. *P*-values are calculated using a Mann-Whitney two-sided test (S1C and S1D Fig).

### Contribution of redundancy and condition dependency to non-responsiveness

Homology-based redundancy pairs had to fulfill the following criteria: the pair is a close paralog pair (either WGD or SSD), both genes are NR and both genes have a condition sensitivity of 5% or less. For calculating the false-discovery rate (FDR), NR-NR close paralog pairs below the sensitivity cutoff were used as true positives and R-R close paralog pairs below the sensitivity cutoff were used as false positives. The lowest false-discovery rate (FDR) was achieved when the applied cutoff was 5% sensitivity (S2 Fig). Pathway-based redundancy pairs had to fulfill the following criteria: the pair has a significant negative genetic interaction score, both genes are NR and both genes have a condition sensitivity of 5% or less. To obtain a similar degree of reliability for pathway-based redundancy estimates, the maximum allowed FDR was set to 10% (as obtained from the homology-based redundancy estimates). In addition to the abovementioned criteria, pathway-based redundancy pairs also have to have less than 40 significant negative genetic interaction scores with other genes in order to obtain a FDR lower than 10%. A pair that isn’t classified as homology-based redundancy or pathway-based redundancy is considered condition dependent.

### Highly regulated genes

Highly regulated genes were determined within the collection of deletion mutants [3]. For each transcript, the observed standard deviation across different deletion mutants or conditions was compared against 1,000,000 permuted transcript profiles. The permuted transcript profiles were generated by randomly selecting expression values within each column (deletion mutant) a million times. *P*-values were calculated as the fraction of permuted profiles with standard deviations equal or larger than the observed standard deviation. Transcripts with *p*-values < 0.05 after multiple testing correction (Benjamini & Hochberg) are considered highly regulated. Wildtype variable genes [3] were excluded from the list of highly regulated genes.

## Supporting information

S1 Fig Sequence divergence in close paralog pairs. **(A)** Ka values as a measurement of sequence divergence are depicted for NR-NR (75), NR-R (83) and R-R (35) pairs. **(B)** Boxplots showing percentage sequence identity across the entire protein length for the same pairs as in A. **(C)** Boxplots showing percentage sequence identity within shared domains for the same pairs as in A. The difference between NR-NR and R-R pairs is statistically significant (*p*-value = 0.008). **(D)** Boxplots showing percentage sequence identity within shared Pfam domains (close paralogs excluded). No significant difference is observed between the three groups. The number of investigated pairs is 96,141, 196,672 and 100,128 for NR-NR, NR-R, and R-R mutants, respectively. Of these pairs, 455, 736 and 320 share at least one Pfam domain and are included in this figure.
(PDF)

S2 Fig Selected sensitivity cutoff for homology based redundancy. Line plot showing the calculated FDR for different sensitivity cutoffs (Materials and Methods). The dashed line indicates the 5% sensitivity cutoff used to identify the contribution of homology-based redundancy to non-responsiveness (FDR ≈ 10%). The same cutoff was also used to infer the contribution of pathway-based redundancy.
(PDF)

S3 Fig Highly regulated protein complex subunits. Line plots showing the mRNA expression changes across 1,484 deletion mutants for protein complexes with a highly regulated subunit (18 in total). Each line shows the expression changes observed for the individual subunits. Subunits highlighted in red have been identified as highly regulated.
(PDF)

S1 Table List of homology-based redundant pairs. (TXT)

S2 Table List of pathway-based redundant pairs. (TXT)

S3 Table List of genes attributed to condition dependency. (TXT)
