# Supplementary figures and images for: Growth condition dependency is the major cause of non-responsiveness upon genetic perturbation
Source: PLoS One. 2017 Mar 3;12(3):e0173432. doi: 10.1371/journal.pone.0173432 (PMC5336285; doi:10.1371/journal.pone.0173432)

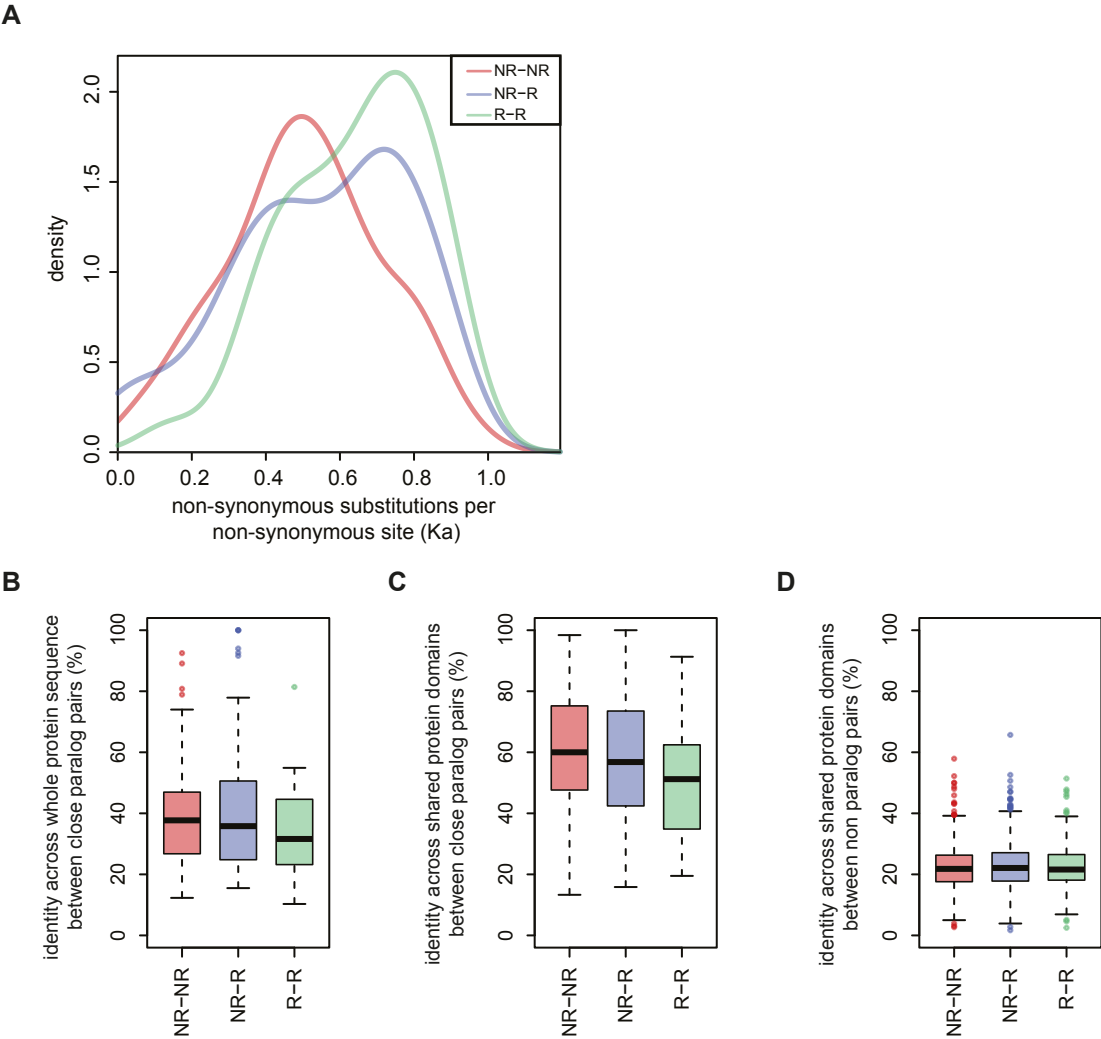

Supplement: S1 Fig — (A) Ka values as a measurement of sequence divergence are depicted for NR-NR (75), NR-R (83) and R-R (35) pairs. (B) Boxplots showing percentage sequence identity across the entire protein length for the same pairs as in A. (C) Boxplots showing percentage sequence identity within shared domains for the same pairs as in A. The difference between NR-NR and R-R pairs is statistically significant (p-value = 0.008). (D) Boxplots showing percentage sequence identity within shared Pfam domains (close paralogs excluded). No significant difference is observed between the three groups. The number of investigated pairs is 96,141, 196,672 and 100,128 for NR-NR, NR-R, and R-R mutants, respectively. Of these pairs, 455, 736 and 320 share at least one Pfam domain and are included in this figure. (PDF) [file pone.0173432.s001.pdf]

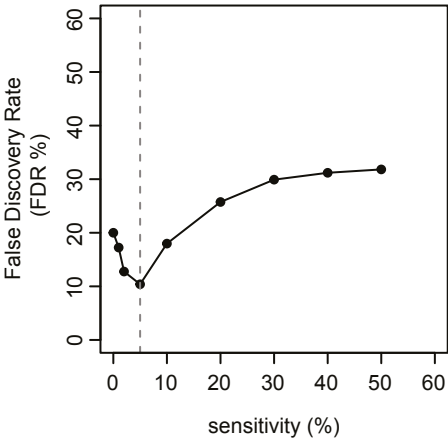

Supplement: S2 Fig — Line plot showing the calculated FDR for different sensitivity cutoffs (Materials and Methods). The dashed line indicates the 5% sensitivity cutoff used to identify the contribution of homology-based redundancy to non-responsiveness (FDR ≈ 10%). The same cutoff was also used to infer the contribution of pathway-based redundancy. (PDF) [file pone.0173432.s002.pdf]

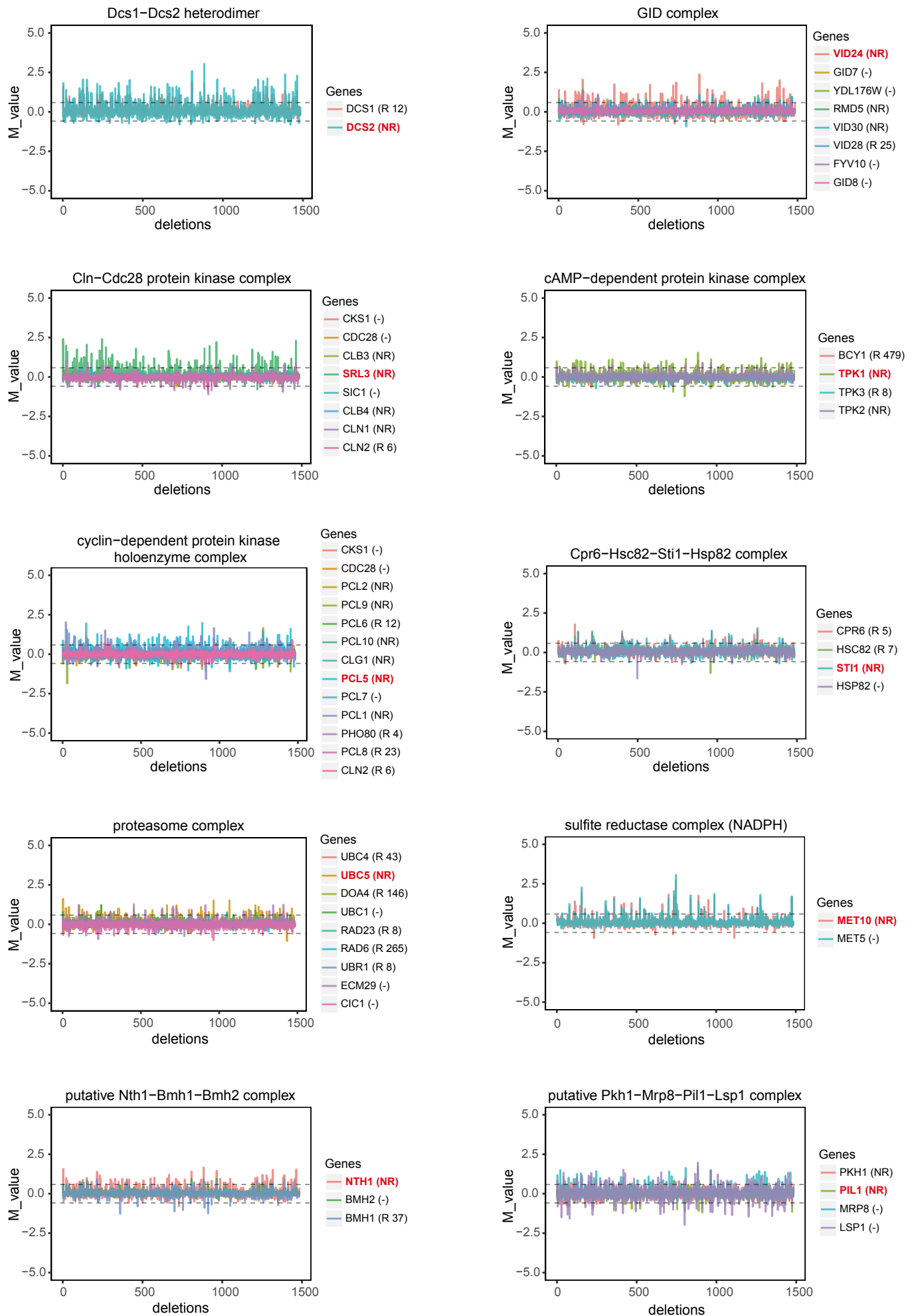

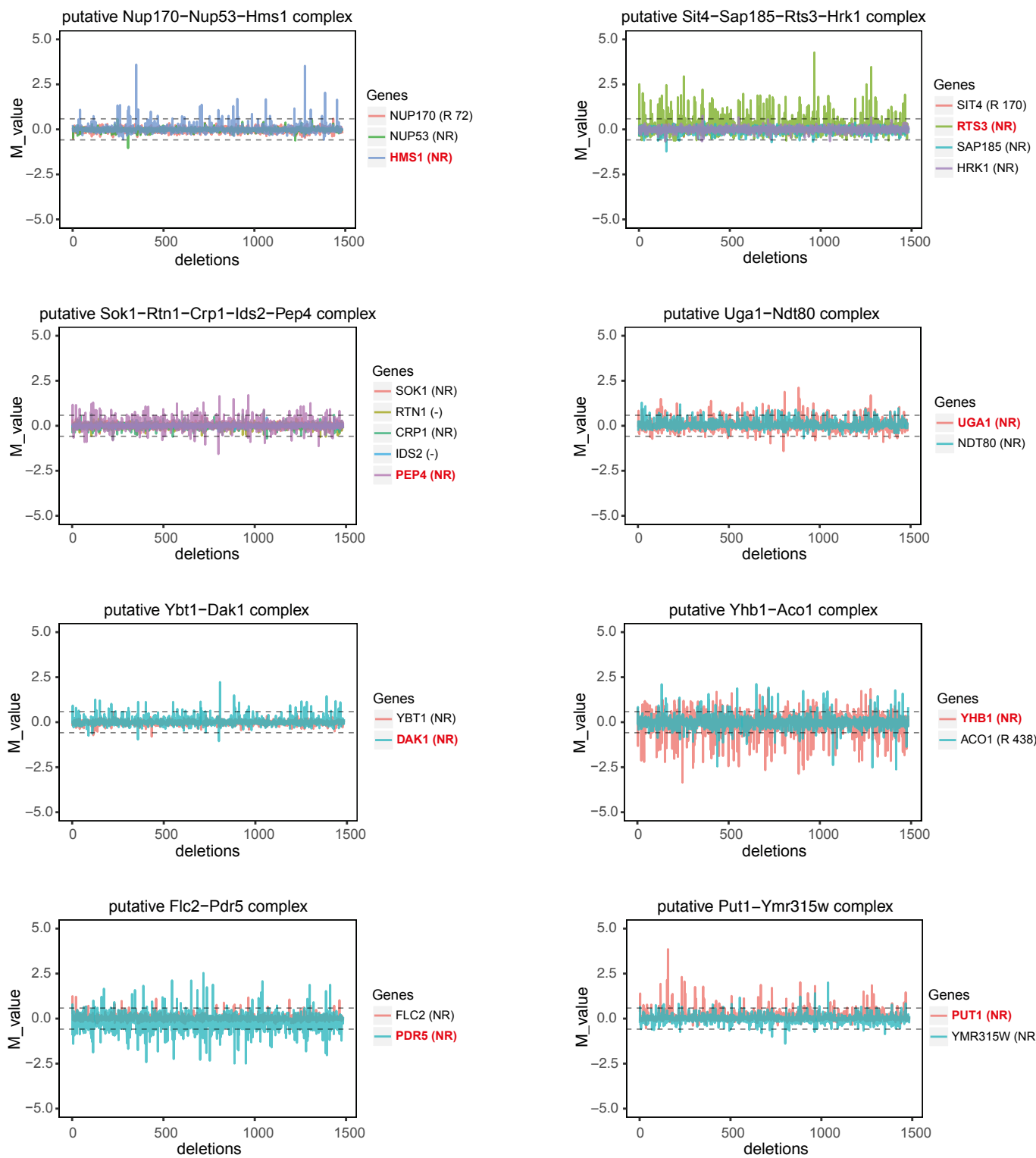

Supplement: S3 Fig — Line plots showing the mRNA expression changes across 1,484 deletion mutants for protein complexes with a highly regulated subunit (18 in total). Each line shows the expression changes observed for the individual subunits. Subunits highlighted in red have been identified as highly regulated. (PDF) [file pone.0173432.s003.pdf]
